# A transvaginal approach to rectovaginal fistulae for the colorectal surgeon: technical notes and case series

**DOI:** 10.1007/s10151-018-1775-4

**Published:** 2018-03-30

**Authors:** R. Bhome, A. Monga, K. P. Nugent

**Affiliations:** 1 Department of Colorectal Surgery, University Hospitals Southampton NHS Trust, Southampton General Hospital, Southampton, SO16 6YD UK; 2 Academic Surgery, University of Southampton, Southampton General Hospital, Level C South Academic Block, Southampton, SO16 6YD UK; 3 Department of Gynaecology, University Hospitals Southampton NHS Trust, Princess Ann Hospital, Southampton, SO16 6YD UK

**Keywords:** Rectovaginal fistula, Repair, Transvaginal, Procedure, Technique

## Abstract

Rectovaginal fistulae (RVF) are not uncommonly seen by the colorectal surgeon and gynaecologist, often debilitating for patients and typically managed with multiple operative procedures, achieving control rather than cure. Transvaginal repair is the least common surgical approach but has clear advantages and equivalent healing rates to other approaches. Here, we describe a simple, safe and effective flapless transvaginal technique for the repair of primary and recurrent low- and mid-level RVF of varying aetiology. We report 15 cases of RVF (nine recurrent) treated by this technique at a single UK centre. The healing rate was 67%. There were no major complications. Median follow-up was 48 months.

## Introduction

Rectovaginal fistulae (RVF) are not infrequently encountered by the colorectal surgeon and gynaecologist. The commonest cause is obstetric injury (prolonged obstructed labour, failed repair of a third or fourth degree perineal tear, or complication of episiotomy). RVF manifest in up to 0.1% of vaginal deliveries in the developed world, with incidence rates of up to 0.3% in developing countries [1]. Crohn’s disease also contributes significantly, with a large UK study showing that greater than 10% of women with Crohn’s disease and an intact rectum develop RVF during the course of their disease [2]. RVF can also occur as a post-surgical complication, typically following low anterior resection, either due to incorporation of the vagina in the anastomotic staple line, or secondary to anastomotic leak. A recent study of over 1400 patients reports an incidence of 1.6% after low anterior resection [3]. A comprehensive list of RVF aetiology is shown in Table 1.

**Table 1:** An aetiological classification of acquired rectovaginal fistula

Broad classification	Sub-classification	Specific condition
Childbirth	Prolonged labour	
	Obstetric injury	3rd/4th degree perineal tear
		Episiotomy
Infection	Local infection	Anorectal abscess Bartholin’s abscess HIV related
	Diverticular disease	
Cancer	Rectal/uterine/cervical/vaginal	
	Irradiation	
Surgery	Anorectal/vaginal surgery	
	Low anterior resection	
	Ileo-anal pouch anastomosis	
	Hysterectomy	
Crohn’s disease		
Other	Faecal impaction	
	Sexual assault	

RVF can be classified by location, size and aetiology. Low fistulae traverse a path between the dentate line of the rectum and the posterior vaginal fourchette, whereas high fistulae open at or near the cervix and mid-level fistulae comprise anything in between. RVF are usually considered simple if less than 2.5 cm, low and caused by obstetric injury or local infection, whereas complex RVF are larger, high, associated with Crohn’s disease, radiation and cancer and often recurrent [4]. Regardless of classification, aetiology is critically important because management of the underlying disease will facilitate any future repair.

**Fig. 1:**
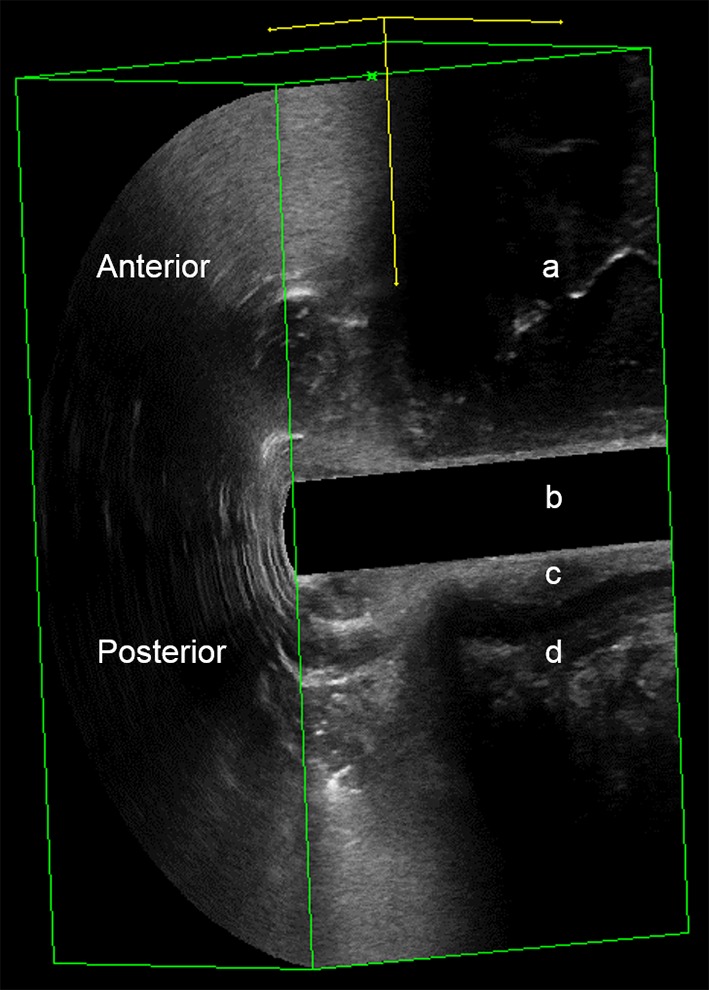
Endoanal ultrasound of low rectovaginal fistula (RVF): *a* RVF tract; *b* ultrasound probe; *c* internal anal sphincter; *d* external anal sphincter

Evaluation of symptomatic patients includes delineation of the tract as well as assessment of the local tissues, including the anal sphincter. The tract is usually identified on examination but in equivocal cases, endoanal ultrasound (for low fistulae; Fig. 1) or cross-sectional imaging (for high fistulae) with magnetic resonance imaging (MRI) or computed tomography may be useful. An assessment of sphincter function is mandatory because it guides the operative approach and is usually assessed by a combination of clinical assessment and ultrasound or MRI.

Managing RVF is notoriously difficult. Conservative measures such as stool bulking and antibiotics will rarely lead to spontaneous healing, although these are advocated initially in benign minimally symptomatic cases [5]. High RVF are best approached transabdominally through laparoscopic or open means, typically resulting in resection of the diseased rectum. Mid-level and low RVF can be approached through the rectum, perineum or vagina. Other reported techniques include Martius flap, gracilis interposition and the use of occlusive biomaterials.

By far the most common approach is transrectal, which typically equates to an endorectal advancement flap. In their meta-analysis of 515 patients with mixed aetiology, Gottgens and colleagues report a 69% healing rate with this procedure [6]. Similarly, the German RVF guidelines estimate healing rates of 50–70% [7]. The transperineal approach has the advantage of allowing simultaneous sphincteroplasty but causes more surgical trauma and leaves a perineal wound. Healing rates range from 35 to 100% [7]. Transvaginal repair is least common, with the fewest studies in the literature, despite the superior surgical access it allows in comparison with a transrectal approach. However, the transvaginal approach is favoured in patients with active Crohn’s disease in the rectum and other studies have shown that endovaginal flaps produce similar outcomes to endorectal flaps [8, 9].

Unfortunately, failure rates are significant with all surgical options for RVF. With both patient and surgeon in mind, we sought to develop a technically simple procedure (with a short learning curve), which is easily reproducible and equally safe and effective as other RVF procedures. Here, we describe a first principles approach to flapless transvaginal RVF repair and report the findings of our initial case series.

## Materials and methods

Consecutive cases of low- and mid-level RVF repaired by a flapless transvaginal technique between 1 April 2012 and 30 September 2017 at University Hospitals Southampton NHS Trust were included. RVF of all aetiologies were included. Cases of RVF repair by alternative techniques were excluded. Defunctioning stoma to treat sepsis had previously been performed where necessary. All procedures were conducted by the same pair of surgeons (colorectal surgeon and gynaecologist).

There was a detailed discussion with each patient about alternatives (including non-surgical options), risks and benefits in the clinic prior to surgery. Local infection (30–40%) and failure to heal (30–40%) were cited as the main risks. Frequencies were quoted based on previous experience and data from our unit.

Patients were admitted on the day of surgery. No bowel preparation was given. All patients had general anaesthesia. Cefuroxime 1.5 g and metronidazole 500 mg were given intravenously at induction. All procedures were conducted in the lithotomy position. Standard skin sterilisation and draping was used. The procedure consisted of the following steps:

1. The fistula tract was identified using a curvilinear fistula probe inserted into the anorectal opening (Fig. 2).

**Fig. 2:**
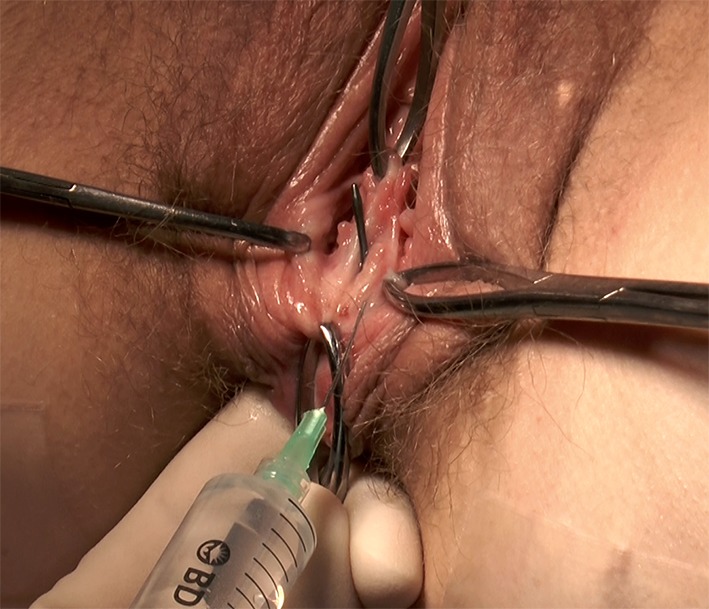
Transvaginal access and local anaesthetic/adrenaline infiltration
2. The vagina was retracted laterally to allow appropriate exposure.
3. A lignocaine (1%)/adrenaline (1 in 200,000) solution was injected circumferentially around the fistula opening on the vaginal side (Fig. 2).
4. Excision of the fistula tract was initiated from the vaginal side with sharp dissection allowing for a 3–4 mm circumferential margin (Fig. 3).

**Fig. 3:**
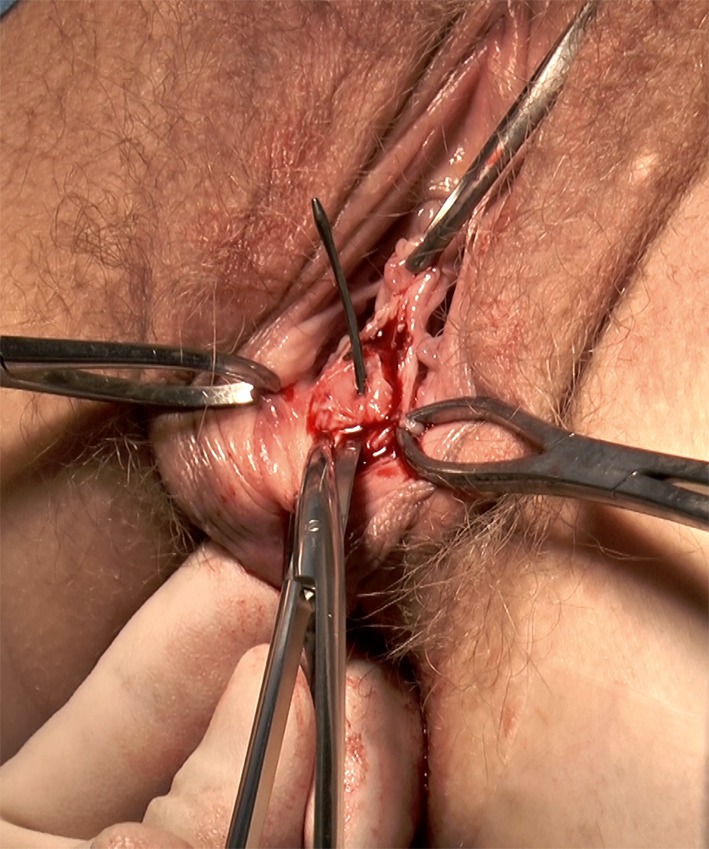
Fistulectomy from the vaginal side
5. The fistula tract, encompassing the vaginal wall, rectovaginal septum and rectal wall, was completely excised.
6. The rectal defect was closed from the vaginal side with one layer of full thickness vertical interrupted sutures, using 3/0 vicryl (Fig. 4).

**Fig. 4:**
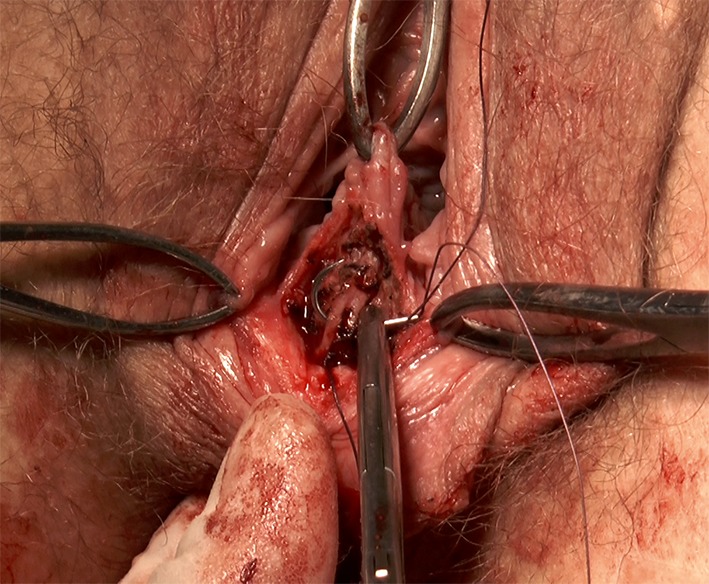
Closure of the rectum
7. The vaginal/septal defect was closed in layers, allowing the rectovaginal septum to be built up as necessary (Figs. 5, 6).

**Fig. 5:**
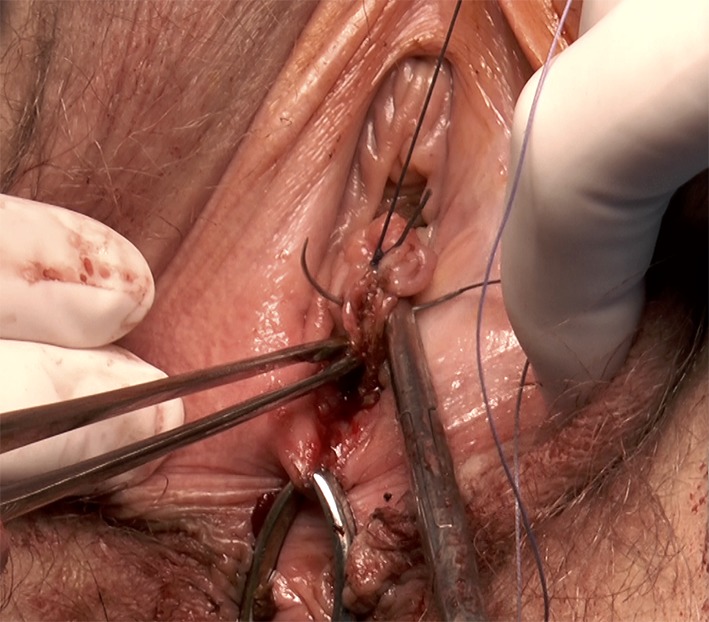
Closure of the vagina and rectovaginal septum

**Fig. 6:**
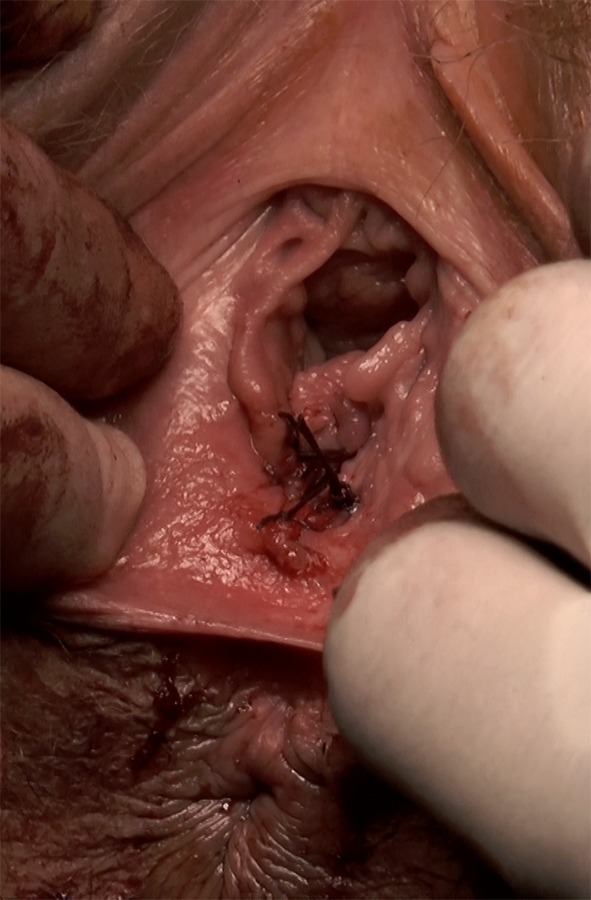
Vaginal appearance immediately following repair
8. A Foley catheter was placed transurethrally into the bladder to aid voiding in the early post-operative period.

A full diet was initiated on recovery from anaesthesia. The urinary catheter was removed on post-operative day 1 in all cases. All patients were prescribed 5 days of oral antibiotic (co-amoxiclav 625 mg t.d.s).

## Results

Fifteen patients were included in the study. All were Caucasian with a median age of 39 years (range 30–70 years). Median American Society of Anaesthetists (ASA) grade was 1 (range 1–3). Eleven patients (73%) were non-smokers. Eight fistulae (53%) had an obstetric cause, with the remainder attributed to Crohn’s disease (*n* = 2), pelvic surgery (*n* = 2), radiation (*n* = 1), cryptoglandular abscess (*n* = 1) and atonic bowel (*n* = 1). Nine patients (60%) had recurrent disease at the time of surgery. Median operating time was 55 min (range 38–90 min) and median blood loss 20 ml (range 0–100 ml). Median length of stay was two nights (range 1–4 nights). There were no major (Clavien–Dindo III–V) complications. Four patients (27%) had grade II complications (wound infection (*n* = 3) and urinary tract infection (*n* = 1)) requiring a single course of oral antibiotics). Healing was achieved in 10 cases (67%). In cases of non-healing (*n* = 5), failure was apparent at a median time of 1 month (range 1–4 months). Of those apparent failures, there was a symptomatic benefit in two (40%) such that no further intervention was required.

In seven out of 15 cases (47%), a diverting stoma was fashioned preoperatively to manage sepsis. Stoma closure was achieved in three of these (43%). Median follow-up for this study was 48 months (range 2–66 months). Table 2 summarises demographic, operative and outcome data.

**Table 2:** Demographic, operative and outcome data of transvaginal flapless RVF repairs between 1 April 2012 and 30 September 2017

Case number	Age (years)	ASA grade	Smoking status	Aetiology	Disease type	Operative time (min)	Blood loss (ml)	Length of stay (days)	Complication	Healing achieved	Time to failure (months)	Pre-operative stoma	Stoma closure achieved	Further intervention required	Follow-up (months)
1	70	3	Non-smoker	Post-surgical: low anterior resection/anastomotic leak	Recurrent	48	0	2	Nil	No	1	Loop ileostomy	No	Yes	66
2	30	3	Smoker	Obstetric: infected episiotomy	Primary	41	0	1	Local infection	No	2	No	NA	Yes	60
3	34	1	Not recorded	Obstetric: fourth degree perineal tear	Primary	44	0	1	Nil	Yes	NA	No	NA	No	56
4	40	1	Non-smoker	Obstetric: fourth degree perineal tear	Recurrent	90	60	3	Nil	Yes	NA	Loop colostomy	Yes	No	55
5	54	1	Non-smoker	Other: Atonic bowel	Recurrent	38	20	2	Nil	Yes	NA	Loop ileostomy	Yes	No	55
6	30	3	Smoker	Obstetric: infected episiotomy	Recurrent	55	100	2	Nil	Yes	NA	No	NA	No	51
7	31	1	Non-smoker	Obstetric: fourth degree perineal tear	Recurrent	60	40	2	Nil	No	4	Loop ileostomy	Yes	No	51
8	31	1	Non-smoker	Obstetric: fourth degree perineal tear	Primary	45	30	1	Nil	Yes	NA	No	NA	No	48
9	37	1	Non-smoker	Cryptoglandular abscess	Recurrent	34	0	2	Local infection	Yes	NA	No	NA	No	45
10	49	1	Non-smoker	Irradiation: anal carcinoma	Primary	60	10	1	Nil	No	1	Loop ileostomy	No	No	32
11	50	1	Not recorded	Obstetric: infected episiotomy	Recurrent	60	0	1	Local infection	Yes	NA	Loop colostomy	No	No	17
12	43	2	Non-smoker	Crohn’s disease	Primary	50	30	1	Nil	Yes	NA	No	NA	No	16
13	38	1	Non-smoker	Post-surgical: rectocele repair	Recurrent	60	10	2	Local infection	No	1	No	NA	Yes	14
14	39	1	Non-smoker	Obstetric: fourth degree perineal tear	Primary	55	50	1	Nil	Yes	NA	No	NA	No	7
15	44	2	Non-smoker	Crohn’s disease	Recurrent	76	50	4	Urinary tract infection	Yes	NA	Loop ileostomy	No	No	2

*RVF* Rectovaginal fistula, *ASA* American Society of Anesthesiologists

## Discussion

Our flapless transvaginal RVF repair technique appears to be safe and effective in primary and recurrent low- and mid-level RVF of varying aetiology. Our healing rate of 67% is equivalent to that reported for endorectal advancement flap [6, 7]. Equally important, there were no major complications.

The procedure described here has an operative time of less than 1 h, which likely reflects the superior surgical access afforded by a transvaginal approach, coupled with the flapless technique. Along with better surgical access, this approach allows reconstitution of the perineal body, enabling wider separation between rectum and vagina, with much less surgical trauma than a perineal approach. Some of the patients in this series had large defects (greater than 2.5 cm) and non-compliant vaginal tissues (post-radiotherapy). In these situations we found that sufficient mobilisation and low tension approximation gave the best chance of healing. Somewhat paradoxically, our unpublished observations suggest that patients having transvaginal repair seem to develop less vaginal scarring than those having other RVF procedures, which may have a beneficial impact on sexual function.

Unfortunately, this case series was not large enough for subgroup analysis to ascertain whether failure was associated with factors such as aetiology, smoking status and pre-operative defunctioning. However, the two patients with Crohn’s disease were both asymptomatic from their RVF at the time of follow-up. Similarly, the patient with post-radiotherapy RVF, although failing to heal, experienced enough symptomatic benefit not to require further intervention. Although we focused on healing as a single primary outcome, we suggest that secondary outcomes such as bowel and sexual function should be included in any future longitudinal studies.

## Conclusions

Transvaginal flapless repair is a safe and effective procedure for low- and mid-level RVF of varying aetiology, which should be considered as a faster and technically less challenging alternative to endorectal advancement flaps by the colorectal surgeon.
